# An A91V SNP in the *Perforin* Gene Is Frequently Found in NK/T-Cell Lymphomas

**DOI:** 10.1371/journal.pone.0091521

**Published:** 2014-03-14

**Authors:** Rebeca Manso, Socorro María Rodríguez-Pinilla, Luis Lombardia, Gorka Ruiz de Garibay, Maria del Mar López, Luis Requena, Lydia Sánchez, Margarita Sánchez-Beato, Miguel Ángel Piris

**Affiliations:** 1 Pathology Department, Fundación Jiménez Díaz, Madrid, Spain; 2 Molecular Pathology Programme, Lymphoma Group, CNIO, Madrid, Spain; 3 Clinical Research Programme, Molecular Diagnostics Clinical Research Unit, CNIO, Madrid, Spain; 4 Clinical Immunology Department, Hospital Clínico de San Carlos, Madrid, Spain; 5 Biotechnology Programme, Monoclonal Antibodies Unit, CNIO, Madrid, Spain; 6 Dermatology Department, Fundación Jimenez Díaz, Madrid, Spain; 7 Biotechnology Programme, Immunohistochemistry Unit, CNIO, Madrid, Spain; 8 Oncology-Haematology Area, Instituto Investigación Sanitaria, Hospital Universitario Puerta de Hierro-Majadahonda, Madrid, Spain; 9 Pathology Department, Hospital Universitario Marqués de Valdecilla, Universidad de Cantabria, IFIMAV, Santander, Spain; Queen's University Belfast, United Kingdom

## Abstract

NK/T-cell lymphoma (NKTCL) is the most frequent EBV-related NK/T-cell disease. Its clinical manifestations overlap with those of familial haemophagocytic lymphohistiocytosis (FHLH). Since *PERFORIN* (*PRF1*) mutations are present in FHLH, we analysed its role in a series of 12 nasal and 12 extranasal-NKTCLs. 12.5% of the tumours and 25% of the nasal-origin cases had the well-known g.272C>T(p.Ala91Val) pathogenic SNP, which confers a poor prognosis. Two of these cases had a double-CD4/CD8-positive immunophenotype, although no correlation was found with perforin protein expression. p53 was overexpressed in 20% of the tumoral samples, 80% of which were of extranasal origin, while none showed *PRF1* SNVs. These results suggest that nasal and extranasal NKTCLs have different biological backgrounds, although this requires validation.

## Introduction

Extranodal NK/T-cell lymphomas (NKTCLs) are the main group of EBV-positive neoplasms that affect TNK cells, according to the recently revised WHO classification, which also includes aggressive NK cell leukaemia, chronic active EBV disease (CAEBV), severe mosquito bite allergy and hydroa vacciniforme. The precise role of EBV in the aetiology of the disease is poorly understood, although the incidence of NKTCL and the other EBV-associated lymphoproliferative disorders (LPDs) parallels the geographic distribution of EBV infection (in Asian and Central and South American populations) [1]. Most NKTCLs probably originate from mature NK cells, while a small proportion of cases, which express αβ or γδ TCR, appear to derive from cytotoxic T-lymphocytes (CTLs). They usually arise as tumours or destructive lesions in the nasal cavity, maxillary sinuses or palate. More rarely, they can appear in other extranodal sites, such as the skin, testis, lung or gastrointestinal tract. Despite their localised presentation in most patients, NKTCL is an aggressive lymphoma associated with a median survival for advanced-stage disease of only 6–12 months. NKTCL has a wide cytological spectrum and is characterised by angioinvasion and angiodestruction, leading to coagulative necrosis. Tumoral cells usually express cytoplasmic CD3, CD2 and, less frequently, CD56, and strongly express cytotoxic markers, including TIA-1, granzyme B and perforin [2], [3], [4].

Perforin is a 67-kDa pore-forming protein that, in mammals, is uniquely expressed in CTL [5], [6]. The complete absence of *PRF1* function typically results in an aggressive, fatal immunoregulatory disorder of early childhood known as type 2 familial haemophagocytic lymphohistiocytosis (FHLH). The overall frequency of *PRF1* mutations in FHLH is between 15% and 50% and depends on the geographical and ethnic origin of the patients [7]. FHLH and EBV-associated haemophagocytic lymphohistiocytosis (EBV-HLH) have overlapping clinical manifestations, whereby CAEBV is often associated with EBV-HLH and some EBV-associated LPD patients eventually evolve into proper NKTCL cases [8], [9]. Interestingly, a case of CAEBV with a mutated *PRF1* gene has been described [10], and a girl initially diagnosed with EBV-HLH carrying a *PRF1* gene mutation (S168N) finally developed an NKTCL [11].

The aim of the study reported in this paper was to establish whether *PRF1* mutations are present in NKTCLs. We analysed a series of 24 consecutive NKTCLs, 12 each of nasal and extranasal origin, and found two *PRF1* single-nucleotide variations (SNVs) in 16.6% of the cases. These SNVs were the well-known pathogenic SNP g.272C>T(p.Ala91Val) and the hitherto unreported c.289G>A(p.Ala97Thr). The p.Ala91Val SNV was present in 12.5% of all cases analysed, which is twice the percentage of cases expected for a Caucasian population (3% in heterozygosity according to http://www.ncbi.nlm.nih.gov/SNP/snp;rs=rs35947132). These data are remarkable, since NKTCLs account for no more than 1% of all lymphomas in Europe. Furthermore, all positive cases were of nasal origin (33.3%), had a peculiar CD4/CD8-positive phenotype, exhibited no correlation with perforin expression, and conferred a poor prognosis on patients (median overall survival of 9.5 months compared with 25.54 and 10.6 months for nasal and extranasal-NKTCLs, respectively). Interestingly, in the present series, p53 was overexpressed in 20% of the tumoral samples, of which 80% were of extranasal origin, and none exhibited *PRF1* SNVs. These data suggest a specific background susceptibility to the development of this subgroup of tumours, at least in the Spanish population. However, a larger series of patients are needed to validate this finding.

## Materials and Methods

### Tissue samples

We analysed a series of 24 consecutive NKTCL cases submitted for diagnosis or a second opinion to the CNIO Pathology Laboratory between 2000 and 2010. Criteria for the diagnosis of NKTCLs were based on the WHO classification [12]. Complete clinical data were obtained from 21 patients. All patients who were alive at the end of the study or the direct relatives of deceased patients provided their written consent to participate. This specific project was supervised and approved by the Ethical Committee of the Hospital Carlos III, Madrid, and Hospital Universitario Marqués de Valdecilla, Santander.

### Tissue microarray construction

Representative areas from formalin-fixed, paraffin-embedded lymphomas were carefully selected on H&E-stained sections and two 1-mm-diameter tissue cores were obtained from each specimen. The tissue cores were precisely arrayed into a new paraffin block using a tissue microarray (TMA) workstation (Beecher Instruments, Silver Spring, MD), following previously described methods [13].

### Immunohistochemistry

TMA sections were immunohistochemically stained using the Endvision method with a heat-induced antigen-retrieval step. Sections were immersed in boiling 10 mM sodium citrate at pH 6.5 for 2 min in a pressure cooker. A panel of eight antibodies (CD3, CD4, CD8, CD56, p53, CD117, beta-catenin and perforin) were analysed (Table 1). Cases were considered positive whether the protein was present in more than 10% of the neoplastic cells. Perforin was analysed with respect to the presence or absence of the protein, the intensity of staining and the pattern of distribution of the granules. Three categories were created based on the intensity of staining: low, intermediate and high. Two groups were recognised, based on the distribution of the granules: a granular pattern limited to the Golgi region or one diffusely distributed throughout the cytoplasm. EBER-positive cells were considered to be neoplastic. Consecutive EBER-positive sections of each case were evaluated to quantify perforin staining. Reactive tonsil tissue was included as a control. The primary antibodies were omitted to provide negative controls.

**Table 1:** Antibodies used in this series.

Antibody	Clone	Source	Dilution	Method (Automated)	Antigen Retrieval
**CD3 FLEX**	Polyclonal Rabbit	Dako	Ready to use	AUTOSTAINER (Dako Cytomation)	TRIS EDTA, 20 MIN
**CD4 FLEX**	4b12	Dako	Ready to use	AUTOSTAINER (Dako Cytomation)	TRIS EDTA, 20 MIN
**CD8 FLEX**	C8/144b	Dako	Ready to use	AUTOSTAINER (Dako Cytomation)	TRIS EDTA, 20 MIN
**CD56 FLEX**	1b6	Dako	Ready to use	AUTOSTAINER (Dako Cytomation)	TRIS EDTA, 20 MIN
**PERFORINA**	5d10	Novocastra	1∶25	AUTOSTAINER (Dako Cytomation)	TRIS EDTA, 20 MIN
**P53**	Do7 (mouse)	Dako	Ready to use	AUTOSTAINER (Dako Cytomation)	TRIS EDTA, 20 MIN
**BETA-CATENIN**	Beta-catenin-1 (mouse)	Dako	Ready to use	AUTOSTAINER (Dako Cytomation)	TRIS EDTA, 20 MIN
**CD117 (C-KIT)**	Rabbit polyclonal	Dako	1/200	AUTOSTAINER (Dako Cytomation)	CITRATE, 20 MIN

### EBV *in situ* hybridisation (EBER)

The presence of EBV RNA was established by non-isotopic *in situ* hybridisation with EBV-encoded RNA (EBER) 1 and 2 oligonucleotide probes (Dakocytomation) in paraffin-embedded tissue sections, as previously described [14].

### *PRF1* gene sequencing

Exons 2 and 3 of the *PRF1* gene have been amplified and sequenced with the primer sets shown in Table 2. Tissue sections from all cases contained between 50% and 80% tumoral cells. The 50-µl PCR reaction volume contained 0.2 mM of each dNTP, 1 unit Taq full DNA polymerase (Clontech Laboratories, Inc.), 0.2 µM of each of the forward and reverse primers and 100 ng of genomic DNA. After initial denaturation at 94°C for 2 min, 35 cycles of 15 s at 94°C, 30 s at 60°C and 30 s at 72°C were run in an MBS Satellite Thermal Cycler (Thermo Scientific). The expected size of the amplified PCR products (Table 2) was determined on an agarose gel with a 100-bp high-range ladder DNA size standard, and the amplified fragments were purified using the MSB Spin PCRapace (Invitek) system and sequenced by means of the dideoxy procedure with the BigDye Terminator v3.1 Cycle Sequencing kit (Life Technologies) and the same primers as for PCR, and analysed with a 3130XL ABI Genetic Analyzer and SeqScape v2.5 software (Life Technologies). PCR products from all cases were sequenced in duplicate from both strands and only cases with changes in both were considered as positive for each different SNV.

**Table 2:** Primers used to amplify most of the exon 2 and 3 coding regions of the *PRF1* gene.

EXON 2	Primer Name	Sequence 5′-3′	Amplicon Length (bp)
	PRF_1_F	CATCCTTCTCCTGCTGCTG	221
	PRF_1_R	CCTCCTGTAGGGCATTTTCA	
	PRF_2_F	GCTCCTTCCCAGTGGACA	265
	PRF_2_R	ACATGCACATTGCTGGTG	
	PRF_3_F	ATCCGCAACGACTGGAAG	241
	PRF_3_R	CTTTCCAGGGCTCCTAGACC	
**EXON 3**	PRF_4_F	TCTCTCTCTTCTCGCAGTTTCC	231
	PRF_4_R	GTCCGTGAGCCCTTCCAG	
	PRF_5_F	CATATCGGCCCTCACTGC	248
	PRF_5_R	GCAGGTCGTTAATGGAGGTG	
	PRF_6_F	GCCTCCTTCCACCAAACCTA	248
	PRF_6_R	GCCCTGTCCGTCAGGTACT	

## Results

### Clinical features

There were 11 males and 13 females, with a median age at diagnosis of 56.6 years (range, 31–89 years). In 12 cases the lesion was initially nasal, two cases each had oropharingeal tumours or involvement of the gastrointestinal tract, six cases involved skin and subcutis, and one case each affected pleura and testis, respectively. We obtained complete clinical data from 21 cases, whose median follow-up was of 18.4 months (range, 1–121 months). None of the patients was referred with haemophagocytic syndrome or leukaemic involvement of the peripheral blood. One patient had previously had a renal transplant, one was HIV-positive and another was HCV-positive. At initial diagnosis the Ann Arbor Stage was low (I–II) in seven and two, and high (III–IV) in five and six nasal and extranasal lymphoma patients, respectively. The frequencies of IPI classes for nasal and extranasal cases at diagnosis were: low (four and two patients, respectively), low-intermediate (four and one), intermediate-high (zero and three) and high (two and two). The frequency across PIT classes at presentation was low (eight nasal and three extranasal cases), low-intermediate (one and four), intermediate-high (one and one) and high (zero and zero). First-line therapy was with CHOP or CHOP-like regimens (CHOP-14, CHOP-21, Mega-CHOP) in 18 patients, followed by local radiotherapy in four patients. Three patients received radiotherapy alone and another one underwent local excision and was administered steroids. Two patients received further autologous stem cell transplantation, and in five cases other second line chemotherapeutic regimens, such as SMILE, alemtuzumab, HPER-CVAD, ESHAP and IEV, were also applied. Seventeen patients died of the disease (nine of nasal and eight of extranasal origin) while four remained alive at last follow-up. The median overall survival for patients with nasal and extranasal NKTCLs was 25.54 (range, 2–121) and 10.6 (range, 1–80) months, respectively (Table 3).

**Table 3:** Most relevant immunohistochemical and clinical data of these series.

CASE	MUTATION	SEX	AD	LDD	P53	CD56	CD8	CD4	PI	PG	STATUS	OS (months)
**1**	NMF	M	77	Nasal tissue	N	HI	N	N	N	NP	A	121
**2**	NMF	M	50	Skin	N	HI	HI	N	Mo	GR	NK	NK
**3**	NMF	M	57	Nasal tissue	N	LI	N	N	L	GR	D	10
**4**	***g.272C>T; Ala91Val***	**M**	**71**	**Nasal tissue**	**N**	**N**	**HI**	**P**	**Mo**	**GR**	**D**	**2**
**5**	NMF	M	47	Skin	N	N	LI	N	L	GR	A	80
**6**	NMF	F	75	Nasal tissue	N	N	N	N	Mo	GR	D	45
**7**	NMF	M	46	Testis	N	LI	N	N	Mo	TC	D	1
**8**	NMF	M	53	Nasal tissue	NV	HI	NV	N	Mo	TC	NK	NK
***9***	*NMF*	*F*	*69*	*Pleura*	*P*	*HI*	*N*	*N*	*Mo*	*TC*	*D*	*2*
**10**	NMF	F	31	Pharynx	N	HI	N	N	H	GR	NK	NK
**11**	NMF	F	53	Nasal tissue	N	N	N	N	L	GR	D	10
***12***	*NMF*	*F*	*43*	*Nasal tissue*	*P*	*HI*	*N*	*N*	*H*	*TC*	*D*	*6*
***13***	*NMF*	*F*	*68*	*Skin*	*P*	*HI*	*N*	*N*	*Mo*	*TC*	*D*	*6*
**14**	NMF	M	78	Nasal tissue	N	HI	N	N	H	GR	A	32
**15**	NMF	F	34	Intestine	NV	N	N	NV	H	GR	D	3
***16***	*NMF*	*F*	*31*	*Stomach*	*P*	*HI*	*N*	*N*	*Mo*	*TC*	*D*	*4*
***17***	*NMF*	*F*	*89*	*Soft tissues*	*P*	*HI*	*N*	*N*	*Mo*	*GR*	*D*	*3*
**18**	NMF	F	62	Oral cavity	N	N	HI	N	L	GR	D	1
**19**	NMF	M	78	Skin	N	LI	HI	N	L	GR	D	5
**20**	***g.272C>T; Ala91Val***	**F**	**48**	**Nasal tissue**	**N**	**HI**	**HI**	**N**	**M**	**TC**	**A**	**21**
**21**	**c.289G>A; pAla97Thr**	**M**	**64**	**Nasal tissue**	**N**	**HI**	**N**	**N**	**M**	**GR**	**D**	**13**
**22**	NMF	F	27	Skin	N	HI	N	N	M	TC	D	1
**23**	***g.272C>T; Ala91Val***	**F**	**52**	**Nasal tissue**	**N**	**HI**	**LI**	**P**	**H**	**GR**	**D**	**2**
**24**	NMF	M	57	Nasal tissue	N	HI	HI	N	H	GR	D	19

NMF:No mutation found; M:Male; F:F; AD:Age at Diagnosis; LDD:Location of the disease at Diagnosis; N:Negative; P:Positive; NV: Not evaluable; HI: High-Intensity; LI:Low-Intensity; L:Low; Mo:Moderate; NP: Not present; NK: Not Known; PI:Perforin Intensity; PG: Perforin Granules; GR: Golgi Region; TC: Troughout the cytoplasm; A: Alive; D:Dead; OS: Overall Survival.

### Immunohistochemical and *in situ* hybridisation characteristics

**Figure 1:**
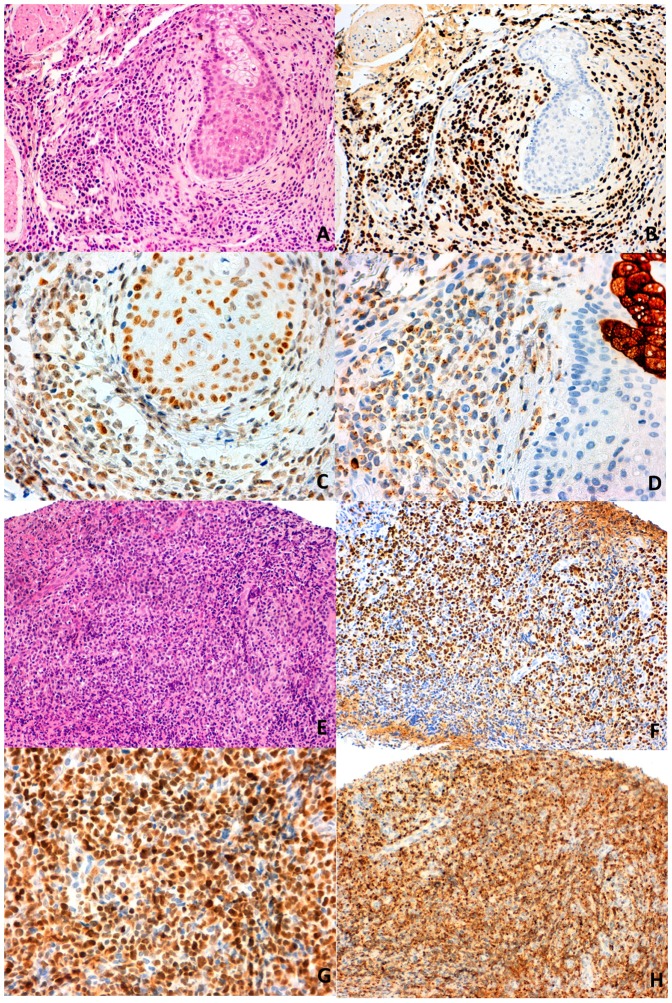
An A91V SNV-positive case (A-HE, staining, B-EBER-positive) showing negativity for p53 (C) and low-level of perforin expression (D). An A91V SNV-negative case (E-HE, staining, F-EBER-positive) showing intense expression of p53 (G) and perforin (H).

EBER and CD3 were positive in all cases, 75% expressed CD56 and there were six CD8-positive, two double-CD8/CD4-positive and 14 double-CD8/CD4-negative cases. Perforin was expressed in 23 (95.8%) of the 24 cases, the intensity of staining being strong in 26.1%, moderate in 43.5% and low in 30.4% of them. Neither CD117 nor beta-catenin was positive in any of the cases, while p53 was intensively positive in five of 22 (22.7%) cases (Table 3 and Figure 1).

### Mutational studies

Three cases were heterozygous for the g.272C>T, p.Ala91Val SNV (12.5%). This is a well-known SNP and the most common base substitution identified in the *PRF1* gene [5]. Interestingly, the substitution of c.289G>A resulting in the amino acid residue change p.Ala97Thr in the *PRF1* gene was found in one other case (2.16%). This SNV was not found in any of the SNP databases (dbSNP137; Hapmap; 1000 genomes) or in the COSMIC database (Table 3). Unfortunately, we did not have normal DNA of the patient to rule out the possibility of it being present in the germline. Additionally, 25% of patients showed other SNPs; four patients had the c822C>T; pAla274Ala SNV (all heterozygous), while six showed the g.900C>T; p.His300His SNV (five heterozygous and one homozygous).

All cases with potential pathological *PRF1* SNVs were of nasal origin, representing 25% (3 of 12) of all nasal cases, or 33.3% when considering all the *PRF1* SNVs. Moreover, three of the four cases with *PRF1* SNV died of the disease, following an overall survival of 9.5 months (range, 2–21 months). These data are more in accordance with the outcome of extranasal than with nasal patients. Two of these cases were those that were double-positive for CD4 and CD8, and one appeared in the tumoral tissue of the renal posttransplant patient. Neither intensity of staining nor the pattern of granule distribution was related to the presence of *PRF1* SNVs.

On the other hand, none of the p53-positive cases showed *PRF1* SNVs, and 80% were of extranasal origin. All cases were double-CD4/CD8-negative, and mainly had a cytoplasmic pattern of perforin expression. All these patients died after a median period of 4.2 months (Table 3).

## Discussion

We report for the first time the presence of the g.272C>T, p.Ala91Val SNV of the *PRF1* gene in NKTCLs. We found it in 12.5% of our cases, which is more than twice the percentage of cases expected in a Caucasian population (3% in heterozygosity according to http://www.ncbi.nlm.nih.gov/SNP/snp;rs=rs35947132). These frequencies are remarkable, since NKTCLs account for no more than 1% of all lymphomas in Europe.

The pathogenic role of the g.272C>T variant has long been controversial [15], [16], although it is accepted that this SNV leads to reduced cytotoxic activity of the perforin protein due to incorrect folding that decreases its cleavage to the active form and increases its degradation [5], [7], [17], [18], [19]. Such a reduction in the level of activity could predispose an individual to late or atypical FHLH, and the development of anaplastic large cell lymphomas, B- and T-cell lymphomas and acute childhood lymphoblastic leukaemia carrying the BCR-ABL fusion gene [7], [20], [21], [22], [23]. Except for the ALCL cases described by Cannella et al [21] none of the other T-cell lymphoma cases exhibiting A91V SNV have been correctly categorised according to the current WHO classification. Cannella et al [21] described this SNV in heterozygosity in eight of the 12 patients in which they found other *PRF1* gene mutations. Clementi et al reported a series of eight cases with equal numbers showing *PRF1* gene mutations in homozygosis and heterozygosity. A91V SNV was heterozygous in three of them, one of the cases being a T-cell non-Hodgkin lymphoma (NHL) [24]. Moreover, a *PRF1* gene mutation was described in two cases of subcutaneous panniculitis-like T-cell lymphoma [24], [25]. One patient presented the 1168C>T (Arg390stop) in a single allele and the other had two mutations, the 272C>T (A91V) and the 1262G>T (Phe 421Cys), in heterozygosity. Both patients suffered from HLH, a widely described phenomenon in both subcutaneous panniculitis αβ and γδ T-cell lymphomas [26].

Except for the double-CD4/CD8 positivity of two cases, which is rare [25], [27], all other immunophenotypes of the cases presented here were in accordance with previous reports [4], [28], [29]. Interestingly, these two double-CD4/CD8-positive cases carried the A91V SNV. In the present study, perforin was present in all but one (95.8%) case, although the pattern and intensity of expression varied from case to case. In the various series reported, perforin-positive cases account for 65–86% of NKTCLs [27], [30], [31], [32], perforin losses being related to poor outcome [27]. In the present study, neither intensity of staining nor the pattern of granule distribution was associated with the presence of the A91V SNV.

The expression of other genes frequently found mutated in cancer and NKTCLs were also investigated in these series. Intense p53 staining was found in 22.7% of cases, while none of them expressed either CD117 or beta-catenin. Interestingly, none of the p53-positive cases showed *PRF1* SNVs, all were double-CD4/CD8-negative, and mainly had a cytoplasmic pattern of perforin expression.

The frequencies of mutations of *p53*, *KIT* and *CTNNB1* genes in NKTCLs varied between Asian countries and the rest of the world, suggesting that the development of these mutations is influenced by geographical, environmental and ethnic differences, as has been discussed by many authors [33], [34], [35], [36], [37], [38]. Moreover, there is a well-known discrepancy in the positive rates between mutational and expression studies [33], [34], [35], [36], [37], [38], [39]. The highest percentages of cases with mutated *KIT* and *CTNNB1* genes have been found in China (11.1%) and Japan (30.0%). Recently, Huang et al were unable to demonstrate nuclear beta-catenin expression, a result that accords with our findings [40]. The highest known rates for p53 mutation are those for Japan and Indonesia, where it was found in 62% and 63% of cases, respectively. The lowest mutation rates have been found in Korea (31% of cases) and Mexico (24%) [3]. Moreover, Quintanilla et al showed that the *p53* mutation is related to large cell morphology and advanced disease stage [35]. Quintanilla et al reported 60% and 86% of p53-positive cases in Mexican and Peruvian populations, respectively [35], [41]. Pongpruttipan et al [29] found 68% of their cases from the Thai population to be positive, while Ye et al [42] found 33.3% of cases to be positive in the Chinese population. The latter two groups exhibited close correlation between p53 expression and both prognosis and advanced disease stage. This and all previously reported studies were done with the same Dako antibody for p53 and using the same cut-off value (>10% positive tumoral cells) to define positivity, with the exception of Ye et al, who used a value of 5%. However, the Quintanilla et al study included exclusively nasal NKTCLs while the others combined nasal and extranasal NKTCL cases in their analyses. In the present series, only one of the five positive cases was of nasal origin, representing 8.3% (one out of 12) of them, while 33.3% of those NKTCLs of extranasal origin (four out of 12) had p53 expression. These data suggest that p53 expression in NKTCLs in the Spanish population is also different from that in other regions of the world. Nevertheless, in considering all the data from the present study, all of the positive cases were III/IV stage patients, while 52.9% and 29.4% of the negative cases were high- and low-stage patients, respectively.

We found the *PRF1* gene A91V SNV in 25% of nasal NKTCLs but in none of the extranasal ones, while 80% of p53-positive cases were extranasal in origin. Several papers have concluded that the clinical features and treatment response of extranasal and nasal NKTCLs are different [43], [44], [45], [46], [47], [48], consistent with the results we present herein. Nevertheless, the underlying features responsible for these differences remain to be determined. On the other hand, some authors have suggested that nasal and extranasal NKTCLs behave alike at the same stage of the disease [46], [47]. One publication addressing this subject has suggested that there are different genetic alterations in the two subgroups [49]. Nasal NKTCL patients with *PRF1* SNVs seem to behave more aggressively than those without it. The median overall survival of nasal NKTCL patients in our series was 25.5 months, while that of patients carrying *PRF1* SNVs was 9.5 months. On the other hand, p53-positive patients died after a median period of 3.7 months (4.2 months when nasal cases were included), while the value for the entire subgroup of extranasal NKTCLs was 10.6 months. Interestingly, only one of the three nasal NKTCLs carrying the *PRF1* SNVs for whom complete clinical data were available had stage IV disease, while the others were stage I and II. In addition, the nasal case and three of the four cases positive for p53 for whom clinical data were complete were stage III and IV, respectively.

The pathogenic role of this *PRF1* SNV has been demonstrated and p53 expression is in most cases a surrogate marker for the *P53* gene mutation. The fact that none of the cases carrying *PRF1* SNVs showed p53 expression suggests the role of different pathways in the two diseases. Considering these findings together, leads us to conclude that nasal and extranasal NKTCLs may have different biological backgrounds. Our series was too short to warrant statistical analysis, so a larger series of patients is needed to confirm our findings.

In conclusion, we suggest that the g.272C>T *PRF1* gene SNV in combination with other gene alterations could increase the risk of developing nasal NKTCL, at least in a subgroup of the Spanish population. Moreover, the presence of p53 in 80% of extranasal NKTCLs not carrying the *PRF1* gene SNV suggests there is a different biological background in nasal and extranasal NKTCLs. However, a larger series of patients, stratified by origin and stage of disease and treated in a uniform way needs to be studied to validate our preliminary findings.
